# Obesity rates in two generations of Swedish women entering pregnancy, and associated obesity risk among adult daughters

**DOI:** 10.1038/srep16692

**Published:** 2015-11-13

**Authors:** José G. B. Derraik, Fredrik Ahlsson, Barbro Diderholm, Maria Lundgren

**Affiliations:** 1 Liggins Institute, University of Auckland, Auckland, New Zealand; 2 Department of Women’s and Children’s Health, Uppsala University, Uppsala, Sweden

## Abstract

We examined changes in obesity rates in two generations of Swedish women entering pregnancy, and assessed the effects of maternal body mass index (BMI) on the risk of overweight or obesity among adult daughters. This study covered an intergenerational retrospective cohort of 26,561 Swedish mothers and their 26,561 first-born daughters. There was a 4-fold increase in obesity rates, which rose from 3.1% among women entering pregnancy in 1982–1988 to 12.3% among their daughters in 2000–2008 (p < 0.0001) when entering pregnancy. The greater the maternal BMI, the greater the odds of overweight and/or obesity among daughters. Underweight mothers had half the odds of having an overweight or obese daughter in comparison to mothers of normal BMI (p < 0.0001). In contrast, the odds ratio of obese mothers having obese daughters was 3.94 (p < 0.0001). This study showed a strong association between maternal obesity and the risk of obesity among their first-born daughters. In addition, we observed a considerable increase in obesity rates across generations in mother-daughter pairs of Swedish women entering pregnancy. Thus, it is important to have preventative strategies in place to halt the worsening intergenerational cycle of obesity.

There is an increasing prevalence of obesity among pregnant women worldwide, in both developed and developing countries^1^. Maternal pre-pregnancy obesity is associated with adverse pregnancy outcomes, such as greater risk of miscarriages, pre-eclampsia, gestational diabetes, and caesarian delivery^2^,^3^. There is also evidence of worse outcomes in the offspring, and a systematic review and meta-analysis concluded that increasing maternal body mass index (BMI) is associated with increased risk of fetal, neonatal, perinatal, and infant deaths^4^. In addition, there is evidence that the fetus of obese mothers develop insulin resistance *in utero*^5^.

A large number of animal studies have shown that maternal obesity during pregnancy is associated with increased obesity risk in the offspring^6^,^7^. As reviewed by Drake & Reynolds^6^, the evidence from animal models has been corroborated by a number of human studies. Research carried out across the world (including Brazil, Finland, Sweden, UK, and USA) have shown that maternal obesity prior to or early in pregnancy is associated with increased adiposity and greater obesity risk in the adult offspring^8^,^9^,^10^,^11^,^12^.

Obesity rates in Sweden have been steadily increasing in both men and women^13^,^14^,^15^. However, there seems to be no data on the possible changes in BMI occurring among women entering pregnancy. As maternal obesity begets obesity in the offspring^16^, such data are of particular interest. Thus, in this study we aimed to evaluate the change in obesity rates in Swedish women entering pregnancy, and to assess the effects of maternal BMI on the risk of overweight or obesity among daughters in adulthood.

## Methods

### Ethics approval

Ethics approval for this study was provided by the Ethics Committee of the Medical Faculty of Uppsala University (Sweden). Study was performed in accordance with approved national and international guidelines. Informed consent was not needed and was therefore not requested from participants, as this is a register-based study where participants were not contacted.

### Study design

The Swedish Birth Register (kept by the National Board of Health and Welfare) was initiated in 1973, and contains data on more than 99% of all births^17^. In Sweden, information is collected prospectively during pregnancy, beginning with the first antenatal visit. This information is then forwarded to the Birth Register, from where data for this study were extracted. All births and deaths are validated every year against the Register of the Total Population (kept by Statistics Sweden), using the mother’s and the infant’s unique personal identification number, which is assigned at birth to each Swedish resident.

This study examined data collected at the first antenatal visit on all Swedish women aged over 18 years, between 1982 and 1988 (i.e. mothers). Data were collected on all their first-born daughters who attended a first antenatal clinic between 2000 and 2008. Daughters were excluded if born to non-Nordic mothers, from multiple births, with congenital malformations, or preterm (< 37 weeks of gestation). In addition, only mothers and daughters aged 18 years or older at the time of first antenatal clinic were included in the study.

Antenatal clinics were mostly during 10–12 weeks of gestation, with 95% of all clinics occurring prior to 15 weeks of gestation. At the first antenatal visit, women were interviewed about current heath, lifestyle, and family history. Weight was measured and current height was self-reported or measured. Women’s BMI were calculated and classified as: underweight <18.5 kg/m^2^, normal weight 18.5–24.99 kg/m^2^, overweight 25.0–29.99 kg/m^2^, and obese ≥30.0 kg/m^2^.

### Statistical analyses

The association between maternal and daughter’s BMI were assessed using multivariate logistic regression models in SPSS version 20 (IBM Corp., Armonk, NY, USA). Models were adjusted for potential confounders, namely age (completed years at delivery), level of education (university degree or not), and smoking habit (daily smoking or not). Models were also run with the inclusion of daughter’s birth weight as a mediator. Rates of different BMI groups across the two generations were compared using chi-square tests in Minitab v.16 (Pennsylvania State University, State College, PA, USA). Age data are means ± standard deviations.

## Results

There were 333,038 Swedish females born in 1982–1988 (daughters), of whom 51,228 gave birth to a first-born child in 2000–2008. Subsequently, daughters were excluded for being born to non-Nordic mothers (2,502) or from mothers younger than 18 years at the first antenatal clinic (632); for being born from multiple pregnancies (616), preterm (2,348), or with malformations (1,737); for being aged less than 18 years (1,500) or carrying a multiple pregnancy (381). Anthropometric data were missing for a further 2,430 daughters and 12,521 mothers. As a result, we studied 26,561 pairs of mothers (aged 26.2 ± 5.0 years) and daughters (aged 22.4 ± 2.3 years).

**Figure 1:**
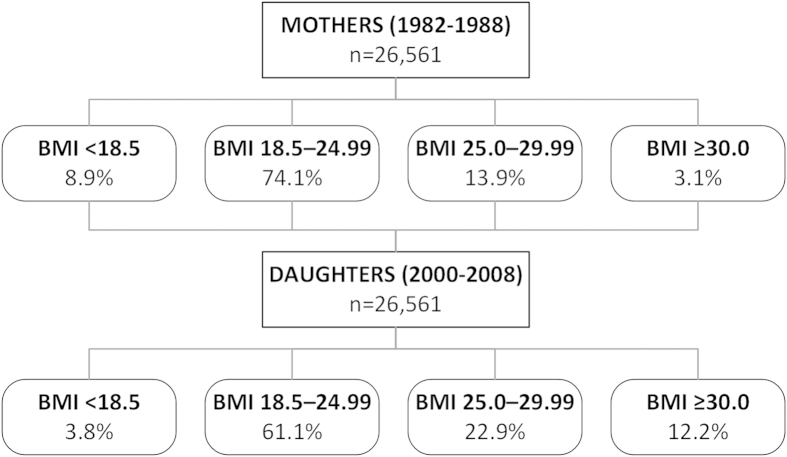
The frequency distribution of mothers and their first-born daughters in Sweden according to BMI (expressed in kg/m^2^).

There was a considerable increase in overweight and obesity rates across the generations, with a concomitant reduction in the rate of underweight women (Fig. 1). Among daughters, 35.1% were overweight or obese, compared to 17.0% of mothers (p < 0.0001; Fig. 1). Further, there was a 4-fold increase in obesity rates, which rose from 3.1% among women entering pregnancy in 1982–1988 to 12.2% among their daughters in 2000–2008 (p < 0.0001; Fig. 1). Of note, during the same period there was a considerable reduction in the number of women smoking, which decreased from 37.8% to 16.4% (p < 0.0001).

There was a strong association between overweight and obesity rates across the two generations, so that the greater the maternal BMI the greater the odds of overweight and/or obesity among the daughters (Table 1). Underweight mothers had half the odds of having an overweight or obese daughter in comparison to mothers of normal BMI (Table 1). In contrast, overweight mothers had odds of having an overweight or obese daughter that were 2.44 greater, and were 2.71 times more likely to have an obese daughter (Table 1). Further, the odds ratio of obese mothers having overweight or obese daughters was 3.71, with the odds of obesity nearly 5-fold greater (Table 1). Unadjusted analyses yielded very similar results (Table 2).

There was a positive but weak correlation between maternal BMI and the birth weight of their daughters (r = 0.18; p < 0.001). Nonetheless, the inclusion of daughter’s birth weight as a mediator into the statistical models had little effect on study outcomes (Table 3).

## Discussion

This study showed that maternal overweight and obesity status was strongly associated with the risk of overweight and obesity among their first-born daughters. This investigation on mother-daughter pairs also showed a 4-fold increase in the rates of obesity across generations among Swedish women entering pregnancy.

Our findings on obesity rates in Swedish women entering pregnancy are not surprising, in light of the increasing rate of obesity among pregnant women globally^1^. In Sweden, a recent study using antenatal clinic data on first-time mothers also showed an increase in the rates of overweight/obesity and obesity early in pregnancy, from 21.2% and 4.5% in 1992 to 32.5% and 10.2% in 2010, respectively^18^. These rates are comparable to the 35.1% and 12.2% rates for overweight/obesity and obesity, respectively, we observed among daughters in 2000–2008. In the wider female population in Sweden, a study in 2,931 women aged 25–34 years in Göteborg showed that 17.9% of women were overweight (c.f. 22.9% in our study) and 8.9% were obese in 2002, with the respective rates for ages 25–64 years being 26.6% and 11.0%^13^. Another study showed that obesity rates in Sweden rose from 8.8% of women in 1981 to 11.9% of women in 1997^15^.

The observed association between mothers’ and daughters’ BMI corroborates the evidence obtained in previous studies. Stuebe *et al.* carried out a large study in the USA on 26,506 women, reporting that high self-reported pre-pregnancy maternal BMI was associated with increased risk of obesity in the daughters in adolescence and adulthood^8^. In Brazil, among 1,443 women, each unit increase in maternal pre-pregnancy BMI was associated with a BMI increase of 0.63 kg/m^2^ in their daughters^10^. In Sweden, a study on 1,103 18-year-old male conscripts reported that maternal pre-pregnancy BMI was positively associated with risk of overweight and obesity in their sons^9^.

Importantly, studies have shown that maternal obesity not only affects the offspring obesity risk, but it also has an adverse effect on their cardiometabolic health in childhood and adulthood^6^. Maternal obesity during pregnancy was associated with increased risk of hospitalization and all-cause mortality in the adult offspring in a large British study on 37,709 people^19^. Thus, our data and those of previous studies strongly suggest that interventions aimed at reducing overweight and obesity rates in women of reproductive age have important implications for subsequent generations. Interestingly, the inclusion of the daughter’s birth weight as a mediator in the statistical models had little impact on study outcomes. This suggests that the effects of maternal obesity on long-term offspring outcomes are not solely associated with its effect on birth weight.

The mechanisms through which maternal obesity affects obesity risk and the long-term health of the offspring are not fully understood, and may include alterations in fetal nutrient supply combined with genetic and epigenetic mechanisms^20^. Lawlor *et al.* speculated that in normal-weight mothers, the effects of maternal weight gain during pregnancy on long-term offspring BMI is mostly explained by shared familial characteristics (genetic and early environmental), whereas in overweight and obese women the evidence indicates mechanisms *in utero*^21^. Heerwagen *et al.* proposed that pre-pregnancy obesity, when combined with normal changes in maternal metabolism, may exacerbate inflammation and blood lipid levels, profoundly affecting the developing embryo and the fetus *in utero*^20^.

A limitation of our study was the lack of birth weight data on the mothers, all of whom were born before the establishment of the Swedish Birth Register in 1973. In addition, the Register does not record paternal anthropometric data, so that the possible associations between fathers’ and daughters’ BMI could not be investigated. Further, as this study only covered first-born daughters, future studies should assess whether a similar trend exist among later-born daughters, especially since we have recently shown that first-borns have greater BMI and increased odds of obesity compared to their second-born sisters in Sweden^22^. Lastly, there were very limited data on maternal birth parameters, so that we were unable to exclude mothers born preterm, from multiple births, or congenital malformations. Nonetheless, this seems to be the largest study yet to investigate obesity risks in mother-daughter pairs, and our well-defined inclusion criteria meant that we were able to investigate a relatively homogeneous cohort.

In summary, although underpinning mechanisms are yet to be elucidated, there is overwhelming evidence that maternal pre-pregnancy overweight or obesity adversely affects the long-term health of the offspring. This study provides further evidence of this relationship, demonstrating a strong association between maternal obesity and the risk of obesity among their first-born daughters. Furthermore, we observed a 4-fold increase in obesity rates across generations in mother-daughter pairs of Swedish women entering pregnancy over a period of approximately 20 years. Thus, it is important to have preventative strategies in place to halt the worsening intergenerational cycle of obesity.

## Additional Information

**How to cite this article**: Derraik, J. G. B. *et al.* Obesity rates in two generations of Swedish women entering pregnancy, and associated obesity risk among adult daughters. *Sci. Rep.* **5**, 16692; doi: 10.1038/srep16692 (2015).

## Figures and Tables

**Table 1:** The association between maternal BMI and first-born daughter’s BMI on 26,561 mother-daughter pairs in Sweden.

		Daughter BMI					
		18.5–24.99 kg/m^2^		≥25.0 kg/m^2^		≥30.0 kg/m^2^	
		OR (95% CI)	p-value	OR (95% CI)	p-value	OR (95% CI)	p-value
Mother BMI	<18.5 kg/m^2^	1.35 (1.22, 1.49)	p < 0.0001	0.52 (0.46, 0.58)	p < 0.0001	0.48 (0.40, 0.58)	p < 0.0001
	18.5–24.99 kg/m^2^	1.00	—	1.00	—	1.00	—
	25.0–29.99 kg/m^2^	0.45 (0.42, 0.49)	p < 0.0001	2.44 (2.26, 2.63)	p < 0.0001	2.71 (2.46, 2.98)	p < 0.0001
	≥30.0 kg/m^2^	0.30 (0.26, 0.35)	p < 0.0001	3.71 (3.18, 4.34)	p < 0.0001	4.65 (3.95, 5.48)	p < 0.0001

Mothers in the normal BMI range (18.5–24.99 kg/m^2^) are the reference group. Data are odds ratios (OR) and associated 95% confidence intervals (CI), adjusted for age, smoking habit, and level of education.

**Table 2:** The association between maternal BMI and first-born daughter’s BMI on 26,561 mother-daughter pairs in Sweden.

		Daughter BMI					
		18.5–24.99 kg/m^2^		≥25.0 kg/m^2^		≥30.0 kg/m^2^	
		OR (95% CI)	p-value	OR (95% CI)	p-value	OR (95% CI)	p-value
Mother BMI	<18.5 kg/m^2^	1.27 (1.16, 1.40)	p < 0.0001	0.55 (0.49, 0.61)	p < 0.0001	0.52 (0.44, 0.63)	p < 0.0001
	18.5–24.99 kg/m^2^	1.00	—	1.00	—	1.00	—
	25.0–29.99 kg/m^2^	0.45 (0.42, 0.48)	p < 0.0001	2.43 (2.27, 2.61)	p < 0.0001	2.69 (2.46, 2.95)	p < 0.0001
	≥30.0 kg/m^2^	0.31 (0.26, 0.35)	p < 0.0001	3.59 (3.10, 4.14)	p < 0.0001	4.52 (3.88, 5.26)	p < 0.0001

Mothers in the normal BMI range (18.5–24.99 kg/m^2^) are the reference group. Results are from unadjusted analyses; data are odds ratios (OR) and associated 95% confidence intervals (CI).

**Table 3:** The association between maternal BMI and first-born daughter’s BMI on 26,561 mother-daughter pairs in Sweden.

		Daughter BMI					
		18.5–24.99 kg/m^2^		≥25.0 kg/m^2^		≥30.0 kg/m^2^	
		OR (95% CI)	p-value	OR (95% CI)	p-value	OR (95% CI)	p-value
Mother BMI	<18.5 kg/m^2^	1.35 (1.23, 1.49)	p < 0.0001	0.52 (0.46, 0.58)	p < 0.0001	0.47 (0.39, 0.58)	p < 0.0001
	18.5–24.99 kg/m^2^	1.00	—	1.00	—	1.00	—
	25.0–29.99 kg/m^2^	0.46 (0.42, 0.49)	p < 0.0001	2.41 (2.23, 2.60)	p < 0.0001	2.66 (2.42, 2.93)	p < 0.0001
	≥30.0 kg/m^2^	0.30 (0.26, 0.35)	p < 0.0001	3.72 (3.18, 4.35)	p < 0.0001	4.56 (3.87, 5.38)	p < 0.0001

Data are odds ratios (OR) and associated 95% confidence intervals (CI), adjusted for age, smoking habit, and level of education, also including daughter’s birth weight as a mediator. Mothers in the normal BMI range (18.5–24.99 kg/m^2^) are the reference group.
